# Characterizing tumor microenvironment heterogeneity in EBV^+^ nTNKL vs ENKTL using spatial transcriptomics and MIF

**DOI:** 10.3389/fimmu.2026.1717844

**Published:** 2026-02-11

**Authors:** Siyu Qian, Zeyuan Wang, Yue Zhang, Wanyue Zhao, Honghan Qiao, Xiaoyan Feng, Yukai Duan, Boyuan Su, Shifeng Hao, Zhenzhen Yang, Mingzhi Zhang, Qingjiang Chen, Guannan Wang, Shenglei Li, Xudong Zhang

**Affiliations:** 1 Department of Oncology, The First Affiliated Hospital of Zhengzhou University, Zhengzhou, China; 2 Academy of First Clinical, Zhengzhou University, Zhengzhou, China; 3 Department of Pathology, The First Affiliated Hospital of Zhengzhou University, Zhengzhou, China; 4 Henan Academy of Innovations in Medical Science, Zhengzhou, China

**Keywords:** EBV - Epstein–Barr virus, EBV+ nTNKL, ENKTL, spatial transcriptomics, TME

## Abstract

**Background:**

Epstein–Barr virus (EBV)-positive nodal T/NK-cell lymphoma (EBV^+^ nTNKL) has recently been delineated in the WHO-HAEM5 classification as a distinct and exceptionally rare entity. Its biology and clinical trajectory remain obscure relative to Extranodal NK/T-cell lymphoma (ENKTL).

**Methods:**

We applied spatial transcriptomics and multiplex immunofluorescence to representative ENKTL and EBV^+^ nTNKL specimens, integrating these data with a retrospective clinical cohort of 14 EBV^+^ nTNKL patients—constituting one of the largest series described to date.

**Results:**

Spatial transcriptomics revealed fundamental differences between ENKTL and EBV^+^ nTNKL. ENKTL, of NK-cell origin, displayed higher malignant cell density, neutrophil enrichment, and an immune-desert phenotype, whereas EBV^+^ nTNKL, of T-cell origin, showed reduced tumor burden, B-cell enrichment, and an immune-active microenvironment with abundant cytotoxic T cells and PD-1/PD-L1 expression. Intercellular communication analyses further highlighted distinct signaling programs—TGF-β/BMP-driven tumor–neutrophil interactions in ENKTL versus CXCL/CCL–GPCR-mediated macrophage crosstalk in EBV^+^ nTNKL. In a retrospective cohort of 14 EBV^+^ nTNKL patients, the disease was frequently complicated by hemophagocytic lymphohistiocytosis and conferred significantly inferior survival, although selected patients achieved durable responses with immune checkpoint inhibitors or CAR-T therapy.

**Conclusion:**

This study delineates the immunologic and molecular architectures of ENKTL and EBV^+^ nTNKL, providing rare insights into this understudied lymphoma. Despite limited sampling, these findings underscore the central role of EBV latency programs and tissue context in shaping tumor ecology and suggest avenues for subtype-tailored therapeutic strategies.

## 1 Introduction

Epstein–Barr virus (EBV), the first human tumor-associated virus, was originally isolated in 1964 from a Burkitt lymphoma cell line (1, 2). EBV infection has been implicated in the pathogenesis of various T/NK-cell lymphoproliferative disorders (TNKL), which predominantly affect individuals in Asian populations, especially those in East Asia. Extranodal NK/T-cell lymphoma (ENKTL) is the most common subtype, marked by its highly aggressive nature and predilection for the midline facial region, manifesting as masses or ulcerations in the nasal cavity, maxilla, and other sites, including the skin and gastrointestinal tract (3, 4). In addition to ENKTL, another EBV-associated TNKL subtype-EBV^+^ nodal TNKL(EBV^+^ nTNKL) has been recognized, presenting exclusively with lymph node involvement. Despite sharing EBV positivity and cytotoxic phenotypes with ENKTL, this subtype exhibits distinct biological features and has been formally recognized as a separate diagnostic category in the 5th edition of the WHO Classification of Hematolymphoid Tumors (WHO-HAEM5) (5, 6).

ENKTL is primarily of NK-cell origin and is characterized by aberrant activation of the JAK-STAT pathway, alongside frequent mutations in epigenetic regulators (BCOR, KMT2D, EP300), tumor suppressor genes (TP53, MGA), and the RNA helicase DDX3X (7, 8). Due to high expression of P-glycoprotein, conventional CHOP-like regimens are largely ineffective, whereas asparaginase-containing regimens such as DDGP and P-Gemox have significantly improved clinical outcomes (9, 10). Emerging therapeutic strategies, including hematopoietic stem cell transplantation and immunotherapy, have also demonstrated promising efficacy (11). In contrast, EBV^+^ nTNKL is predominantly of T-cell origin and is characterized by lower genomic instability and recurrent mutations in TET2, PIK3CD, DDX3X, and STAT3 (7). This lymphoma subtype exhibits a highly aggressive clinical course. Currently, no standardized treatment guidelines exist, and therapeutic strategies are largely extrapolated from ENKTL management. While both subtypes are driven by EBV infection and share cytotoxic features (GZMB^+^, TIA1^+^), they exhibit substantial intrinsic heterogeneity (12, 13).

The tumor microenvironment (TME) constitutes a critical ecosystem for tumor cell survival, comprising malignant cells, stromal cells, and immune cells. Through complex molecular and cellular interactions, the TME plays a pivotal role in driving tumor heterogeneity. Chronic EBV infection has been shown to remodel the TME, facilitating immune evasion and tumor progression (14, 15). However, the specific TME alterations in these two distinct lymphoma subtypes remain poorly understood. To bridge this knowledge gap, we aim to systematically dissect the TME landscapes of these two distinct EBV-associated lymphomas using multiplex immunofluorescence (MIF) and spatial transcriptomics. Our study seeks to unravel their distinct immune architectures and cellular ecosystems, thereby providing new insights into their pathobiology and informing rational therapeutic strategies for EBV^+^ nTNKL.

## 2 Materials and methods

### 2.1 Study group

Tumor tissue samples were obtained from 11 patients with ENKTL and 11 patients with EBV^+^ nTNKL. All cases were pathologically confirmed by expert hematopathologists at the First Affiliated Hospital of Zhengzhou University. The diagnosis of EBV^+^ nTNKL required fulfillment of the following criteria: (1) cytotoxic T-cell or NK-cell lymphoma (TIA1^+^ and GZMB^+^); (2) EBER positivity in at least 50% of the tumor cells; (3) primary involvement of the lymph nodes; and (4) exclusion of T/NK-cell lymphoproliferative disorders associated with immunodeficiency, extranodal NK/T-cell lymphoma, and lymph node involvement secondary to systemic EBV^+^ T and NK-cell lymphoma in childhood. This study was approved by the Ethics Committee of the First Affiliated Hospital of Zhengzhou University and conducted in accordance with the principles of the Declaration of Helsinki (2024-KY-2210). Written informed consent was obtained from all patients.

### 2.2 Spatial transcriptomic sequencing

FFPE TNKL tissue blocks were sectioned at 5 μm for spatial transcriptomic (ST) profiling using the 10× Genomics Visium platform. RNA quality was assessed by DV200 analysis following RNA extraction, and only samples with DV200 ≥50% were included. Tissue adhesion testing was performed to ensure section integrity. Sections underwent deparaffinization, antigen retrieval, and crosslink reversal, followed by whole-transcriptome probe hybridization and ligation. After permeabilization, ligated products were captured by spatially barcoded oligonucleotides on Visium slides to generate spatially resolved libraries, which were sequenced on an Illumina NovaSeq 6000 platform. Sequencing data were aligned and quantified using Space Ranger (v2.0.0) to generate spatial gene expression matrices. Approximately 180 million reads were obtained per sample, corresponding to an average depth of ~60,000 reads per spot. Filtered feature-barcode matrices were processed in Seurat (v5.1.0) using R (v4.4.2). Spots with fewer than 100 detected UMIs were excluded, and background spots outside tissue regions were manually removed for the EBV^+^ nTNKL sample. No filtering based on mitochondrial or ribosomal gene content was applied. The three samples were normalized separately and subsequently integrated to correct for batch effects. Quality control metrics are summarized in **Supplementary Table S1**.

### 2.3 Spatial transcriptomic analysis

Standardized workflows were applied for quality control and downstream analysis of spatial transcriptomic data. Initially, expression matrices generated by Space Ranger were preprocessed via the Seurat package (v5.1.0) in R (v4.4.2). SC Transform normalization was then applied, and the top 3000 highly variable genes were selected for subsequent analyses. Principal component analysis was performed on the normalized data, and the top 30 principal components were used for downstream clustering. Spots were clustered via the Find Neighbors and Find Clusters functions with a resolution of 0.4. Differentially expressed genes (DEGs) for each cluster were identified using the Wilcoxon test via the Find All Markers function. Cell type annotation was conducted based on cluster-specific marker genes via the Cell Marker 2.0 database, Cell Taxonomy, and relevant literature. DEGs, defined as genes with adjusted p values < 0.05 and average log2-fold changes > 0.3, were analyzed for functional enrichment via the cluster Profiler package (v3.10.1), including Gene Ontology (GO) and Kyoto Encyclopedia of Genes and Genomes (KEGG) analyses.

#### 2.3.1 Identification of malignant cells

Copy number variations (CNVs) in spatial transcriptomic data were inferred using the inferCNV R package (v1.12.0). A normal control sample (chronic rhinosinusitis, CRS; case 1) was used as the diploid reference. Genes with average expression <0.1 or detected in fewer than 10 spots were excluded. CNV profiles were smoothed along the genome using a sliding window of 101 genes with subsequent denoising. CNV scores were calculated for each spatial spot to quantify large-scale chromosomal gains or losses relative to the reference. Spots exhibiting elevated CNV scores (>1800 in this dataset) and forming distinct clusters from the reference group during hierarchical clustering (Ward.D2) were classified as malignant.

#### 2.3.2 Calculation of enrichment scores of cell subpopulations

Marker gene sets for specific cell types were obtained from the Cell Marker 2.0 and Cell Taxonomy databases. The enrichment scores for each gene set within individual samples were calculated via the Add Module Score function.

#### 2.3.3 Pseudotime trajectory analysis

Pseudotime trajectory analysis was performed using Monocle2 (v2.34.0) to infer continuous developmental trajectories and state transitions of selected cell populations. Lineage-specific analyses were conducted for malignant and normal NK cells in ENKTL samples, as well as cancer-associated macrophages and malignant T and NK cells in the EBV^+^ nTNKL sample. After normalization and dispersion estimation, ordering genes were identified using the differentialGeneTest function with a significance threshold of q < 0.05. Dimensionality reduction and trajectory reconstruction were performed using the DDRTree algorithm. Cells were then ordered along the reconstructed trajectory using the orderCells function to generate pseudotime progression. Genes exhibiting significant expression dynamics along pseudotime were identified using differentialGeneTest, and branch-specific gene regulation at trajectory bifurcation points was further characterized using branch expression analysis modeling.

#### 2.3.4 Cell–cell communication analysis

Cell–cell communication was analyzed using CellChat (v2.0.0) by integrating normalized expression matrices, spatial coordinates, and cell-type annotations from ENKTL and EBV^+^ nTNKL samples. Intercellular communication probabilities were inferred using the computeCommunProb function with the truncatedMean method (trim = 0.1) based on the Secreted Signaling subset of the CellChatDB.human database, with a minimum threshold of 10 cells per interacting cell type (min.cells = 10). Signaling pathway–level communication activities were computed using computeCommunProbPathway. Communication networks and signaling roles were visualized using netVisual_heatmap and netAnalysis_signalingRole_scatter.

### 2.4 Multiplex immunofluorescence analysis

Formalin-fixed, paraffin-embedded (FFPE) tissue samples were sectioned at a thickness of 3 μm. Antigen retrieval was performed via either heat-induced epitope retrieval or enzymatic digestion, depending on the target antigen. The tissue sections were then blocked with blocking buffer containing bovine serum albumin to reduce nonspecific binding. Primary antibodies targeting specific antigens were incubated at the appropriate temperature for 1–2 hours or overnight, followed by washes with phosphate-buffered saline to remove unbound antibodies. Detailed antibody information is provided in **Supplementary Table S2**. The sections were incubated with fluorophore-conjugated secondary antibodies in the dark to prevent photobleaching. Nuclei were counterstained with DAPI or Hoechst dye, and samples were mounted with anti-fade mounting medium. Fluorescence imaging was performed via fluorescence or confocal microscopy, enabling visualization and analysis of antigen distribution across multiple fluorescence channels. For quantitative analysis, an independent validation cohort comprising samples from 10 ENKTL and 10 EBV^+^ nTNKL patients was analyzed. For each sample, two randomly selected regions of interest were evaluated. Fluorescence imaging was performed using fluorescence or confocal microscopy, enabling visualization and quantitative analysis of antigen distribution across multiple fluorescence channels.

### 2.5 Clinical data collection

From January 2015 to December 2024, 14 patients with EBV^+^ nTNKL were diagnosed through clinical, laboratory, and pathological evaluations. In addition, 20 patients with ENKTL were randomly selected, and both groups were enrolled in this study at the First Affiliated Hospital of Zhengzhou University. Patient data were collected from medical records and telephone follow-ups, with the follow-up period ending on March 31, 2025. T-cell receptor (TCR) gene rearrangement analysis was performed as part of routine clinical diagnosis using multiplex PCR with capillary electrophoresis on FFPE tissue. Rearrangements of TCRβ (Vβ–Jβ and Dβ–Jβ), TCRδ (Vδ–Dδ–Jδ), and TCRγ (VγI–Vγ10–Jγ and Vγ9–Vγ11–Jγ) loci were assessed, and clonality was defined by the presence of a dominant peak within the expected size range, whereas polyclonal patterns showed Gaussian or multiple peaks. TCR results were used for diagnostic support only. Treatment efficacy was assessed according to the 1999 Cheson lymphoma criteria and categorized as complete remission (CR), partial remission (PR), stable disease (SD), or progressive disease (PD). Progression-free survival (PFS) was defined as the time from the initiation of treatment until disease progression, death from any cause, or the follow-up cutoff date. Overall survival (OS) was defined as the time from treatment initiation to death from any cause or the follow-up cutoff date.

### 2.6 Statistical analysis

Statistical analyses were conducted via SPSS software (version 21.0). Independent samples t tests were used to compare patient age between the two groups, whereas chi-square tests were used to assess differences in sex, Lugano stage, IPI score, B symptoms, EBV DNA levels, incidence of hemophagocytic syndrome, and rates of CD8/CD56 positivity. Comparisons of MIF–based quantitative data between ENKTL and EBV^+^ nTNKL were performed using two-tailed Student’s t tests. A P value < 0.05 was considered statistically significant. Kaplan–Meier survival curves were constructed for both groups, and the log-rank test was performed (R 4.2.1). A P value < 0.05 was considered statistically significant.

## 3 Results

### 3.1 Spatial transcriptomics reveals the configuration of the microenvironment in ENKTL and EBV^+^ nTNKL

**Figure 1:**
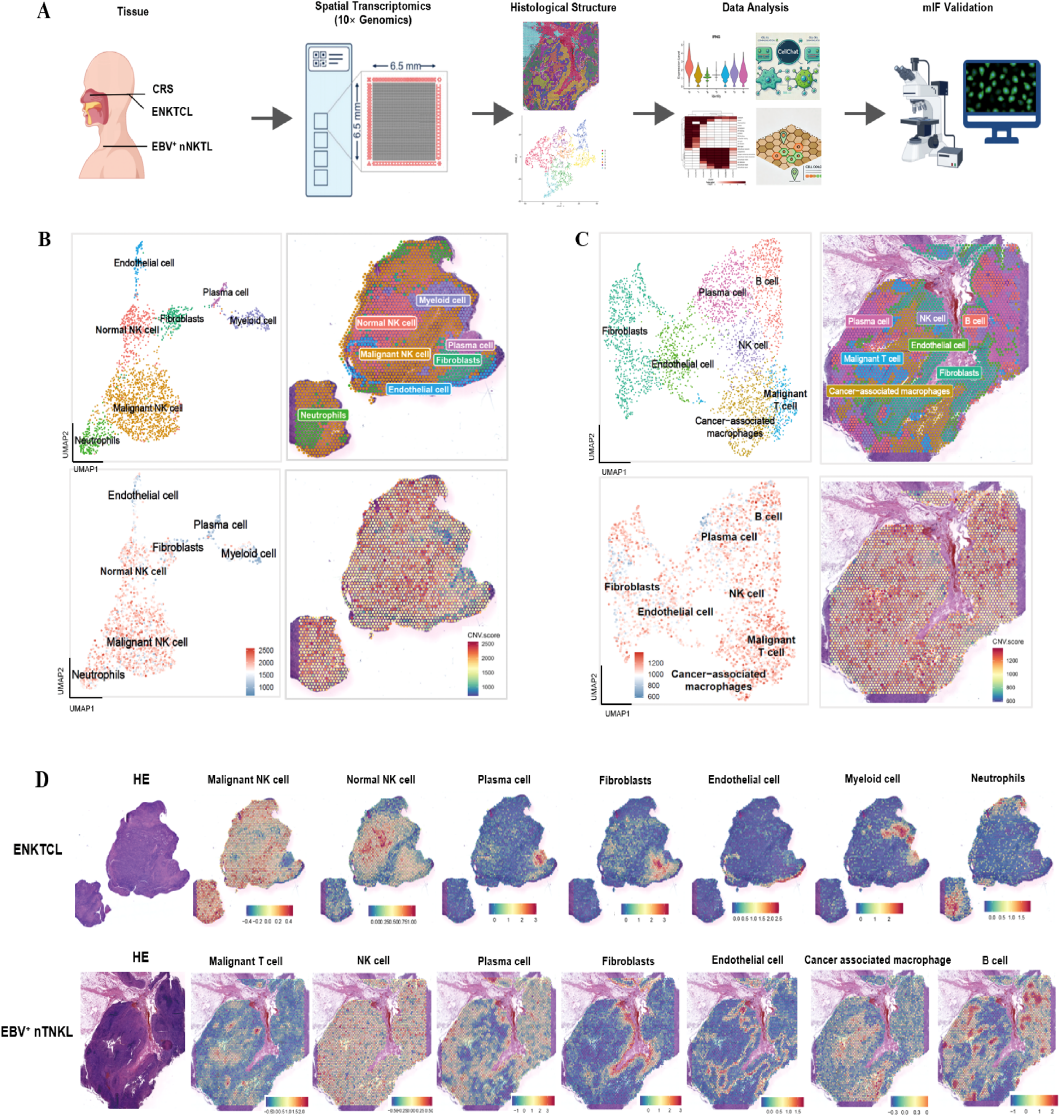
Configuration of the TME in ENKTL and EBV^+^ nTNKL. **(A)** Schematic overview of the study including sample collection, spatial transcriptomics sequencing and MIF. **(B, C)** U-MAP plot and spatial mapping diagram displaying all cell types of ENKTL **(B)** and EBV^+^ nTNKL **(C)**: Top left: U-map plot, Top right: The results of clustering are overlaid onto spatial spots, Bottom left: Distribution of aneuploid cells in U-MAP plot, Bottom right: The CNV results overlaid onto spatial spots. **(D)** Spatial expression of the marker genes.

To comprehensively profile the tumor microenvironment and spatial architecture, we conducted spatial transcriptomic analysis via the 10× Genomics Visium platform on FFPE tissue sections from three individuals, including one patient with EBV^+^ nTNKL, one patient with ENKTL, and one patient with CRS as a control. The clinicopathologic characteristics of these three cases are summarized in **Table 1**. The ENKTL sample originated from the nasopharynx and exhibited an NK-cell immunophenotype, whereas the EBV^+^ nTNKL sample involved lymph nodes and showed a T-cell lineage, as supported by TCR rearrangement analysis. Both malignant cases were EBV-positive and expressed cytotoxic markers, including TIA1 and GZMB, while the CRS sample served as a reference for non-neoplastic inflammatory tissue architecture. Tissue sections from these three samples were mounted on ST microarray slides containing spatial barcodes for subsequent analysis. The detailed experimental workflow is shown in **Figure 1A**. Following quality control filtering, we obtained 2683, 2215, and 4446 specific captured areas (spots) from the ST microarray slides of patients 1, 2, and 3, respectively. The corresponding gene counts detected were 18,007, 17,982, and 18,121, respectively. Each spatial spot measured approximately 55 μm in diameter and encompassed multiple cells, potentially reflecting a mixture of diverse cellular populations. These complex tissue regions were subsequently subjected to computational deconvolution and visualized individually.

**Table 1:** Clinical features of the three samples.

Patient	Diagnosis	Tumor location	Immunohistochemistry						TCR rearrangement
			CD3	CD8	CD56	TIA1	GZMB	EBER	
Case1	CRS	**/**	**/**	**/**	**/**	**/**	**/**	**/**	**/**
Case2	ENKTL	Nasopharynx	**+**	Scattered**+**	**+**	**+**	**+**	**+**	**-**
Case3	EBV^+^ nTNKL	Lymph nodes	**+**	**+**	Scattered**+**	**+**	**+**	**+**	**+**

Dimensionality reduction and unsupervised clustering identified seven distinct cellular clusters in both the ENKTL and EBV^+^ nTNKL samples. Cluster annotation was performed by integrating canonical lineage markers, copy number variation (CNV) profiles, and reference signatures from previously published single-cell transcriptomic datasets of NK/T-cell lymphomas (**Figures 1B, C**; **Supplementary Figures S1A, B**). Specifically, clusters exhibiting high expression of KLRC4, GZMA, NKG7, and GZMM were annotated as NK-cells; clusters with CXCL8 and G0S2 enrichment were classified as neutrophils; those expressing COL1A1, COL3A1, FBLN1, and APOD were identified as fibroblasts; and clusters with elevated levels of KRT19, HSPB1, and ECSCR were designated as epithelial cells. Myeloid cells were characterized by the expression of LYZ, IDO1, and TAP1; plasma cells, by IGHA1, IGKC, and IGHG1; cancer-associated macrophages, by EGFL6H, SPP1, and ADGRE1; and B cells, by CD79A and CD79B. CNV analysis further substantiated the malignant identity of specific clusters (**Figures 1B, C**; **Supplementary Figures S1A, B** for details). Spatial projection demonstrated that these annotated cell populations displayed region-specific distributions concordant with histopathological features (**Figure 1D**).

Both ENKTL and EBV^+^ nTNKL samples harbored diverse stromal and immune cell types, including NK-cell, fibroblasts, plasma cells, myeloid cells, and endothelial-like cells. Notably, a neutrophil cluster was uniquely observed in ENKTL, whereas a B-cell cluster was specifically enriched in EBV^+^ nTNKL. This distinct enrichment likely reflects their tissue origins, with ENKTL arising predominantly in the nasal cavity, often associated with chronic rhinitis, and EBV^+^ nTNKL occurring in lymph nodes enriched in B-cell populations. To further investigate functional heterogeneity, we performed gene set enrichment analysis on the top DEGs from each cluster (**Supplementary Figures S1C, D**). In ENKTL, the neutrophil cluster was enriched for pathways associated with immune system activation, whereas in EBV^+^ nTNKL the B-cell cluster was enriched for pathways related to activation and proliferation.

### 3.2 Heterogeneity of malignant cells in ENKTL and EBV^+^ nTNKL

**Figure 2:**
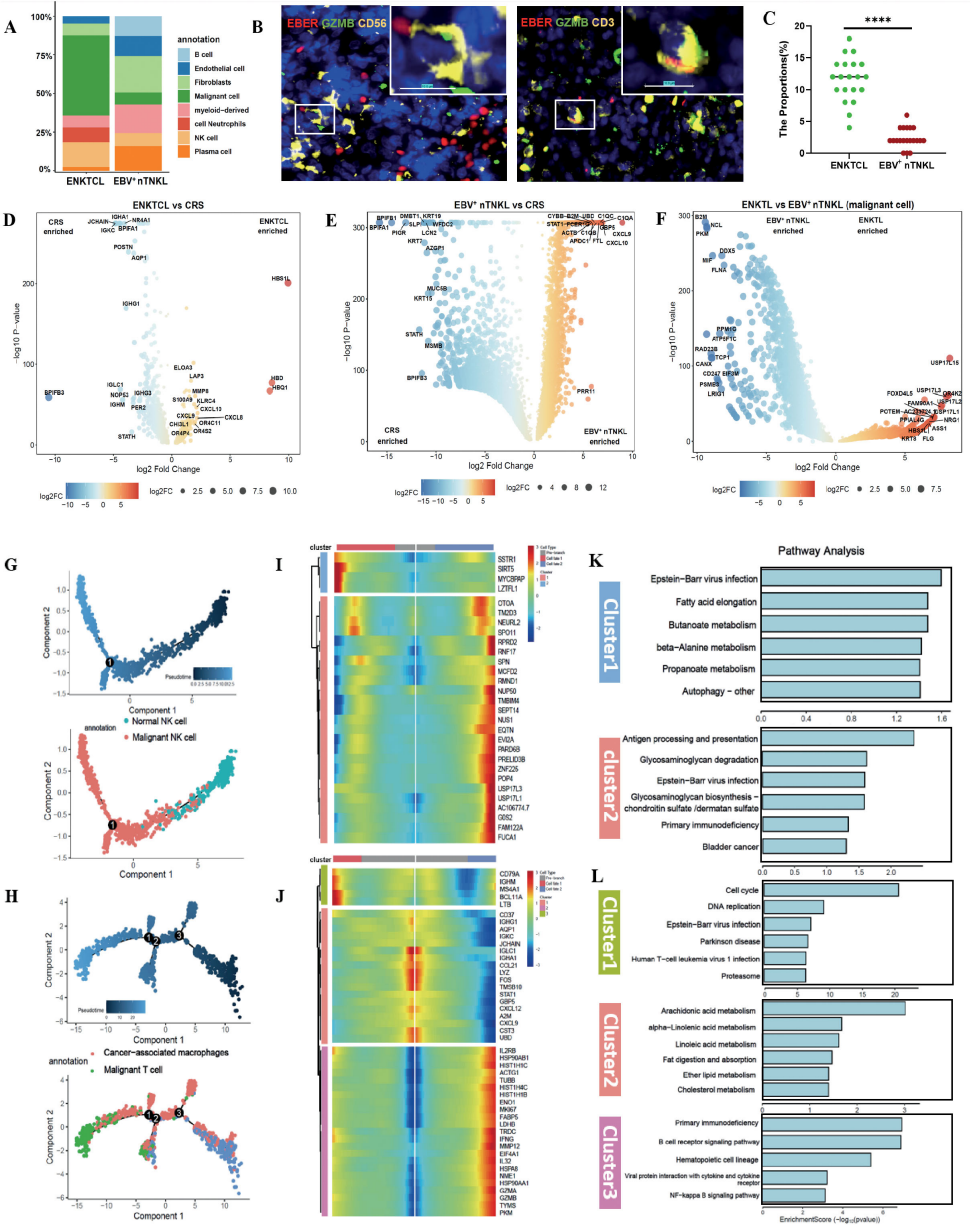
Distinct patterns of malignant cell heterogeneity in ENKTL and EBV^+^ nTNKL. **(A)** Number of spot counts and the percentage of each cluster in individual tissues. **(B)** MIFIF of CD56^+^GZMB^+^EBER^+^ malignant cells in ENKTL and CD3^+^GZMB^+^EBER^+^ malignant cells in EBV^+^ nTNKL(Scale bar=10μm). **(C)** Quantitative analysis of tumor cell densities in ENKTL and EBV^+^ nTNKL. **(D–F)** Differential gene expression analysis of malignant cells: **(D)** ENKTL vs CRS, **(E)** EBV^+^ nTNKL vs CRS, and **(F)** ENKTL vs EBV^+^ nTNKL. **(G, H)** Pseudotime trajectory analysis of malignant cells from ENKTL **(G)** and EBV^+^ nTNKL **(H)**. **(I, J)** Differentially expressed genes along the pseudotime trajectory in ENKTL **(I)** and EBV^+^ nTNKL **(J)**. **(K, L)** KEGG pathway enrichment analysis of pseudotime-associated genes in ENKTL **(K)** and EBV^+^ nTNKL**(L)**.

By quantifying the number of spatial transcriptomic spots annotated as malignant, we compared the malignant cell density between ENKTL and EBV^+^ nTNKL and found a significantly greater density in the ENKTL samples (**Figure 2A**). To validate this observation, we established an independent cohort comprising samples from 10 ENKTL and 10 EBV^+^ nTNKL patients, with two randomly selected regions analyzed per sample. Malignant cells, identified by MIF staining with EBER/GZMB/CD56 for ENKTL and EBER/GZMB/CD3 for EBV^+^ nTNKL, were significantly more abundant in ENKTL than in EBV^+^ nTNKL in the validation cohort, consistent with the spatial transcriptomic results (**Figures 2B, C**). To further investigate the transcriptomic features of the malignant cell clusters, we conducted differential gene expression (DEG) analysis. Compared with CRS tissue, EBV^+^ nTNKL showed increased expression of B-cell– and immune regulation–associated genes (AQP1, IGHA1, NR4A1), a feature not observed in ENKTL(**Figures 2D–F**). KEGG pathway enrichment analysis revealed activation of neuroactive ligand–receptor interaction, cytokine–cytokine receptor interaction, and calcium signaling pathways in ENKTL, whereas dysregulated chemokine signaling and lysosomal pathways were enriched in EBV^+^ nTNKL (**Supplementary Figures S1E–G**).

TCR rearrangement analysis indicated that malignant cells in ENKTL originated from NK-cell, whereas those in EBV^+^ nTNKL were derived from T cells. Monocle-based trajectory inference corroborated these lineage origins, and pseudotime analysis delineated gene expression dynamics across malignant progression (**Figures 2G–J**). Unsupervised clustering of pseudotime-associated genes, followed by KEGG enrichment, identified transcriptional programs linked to malignant transformation (**Figures 2K, L**). In ENKTL, malignant cells exhibited sequential activation of pathways related to EBV infection, fatty acid elongation, butanoate metabolism, and antigen processing and presentation, whereas in EBV^+^ nTNKL, they showed dysregulation of pathways involving cell cycle control, DNA replication, EBV infection, and arachidonic acid metabolism. These findings point to subtype-specific trajectories and pathway dependencies that may shape their tumor microenvironment.

### 3.3 Distinct functional patterns of immune cells in ENKTL and EBV^+^ nTNKL

**Figure 3:**
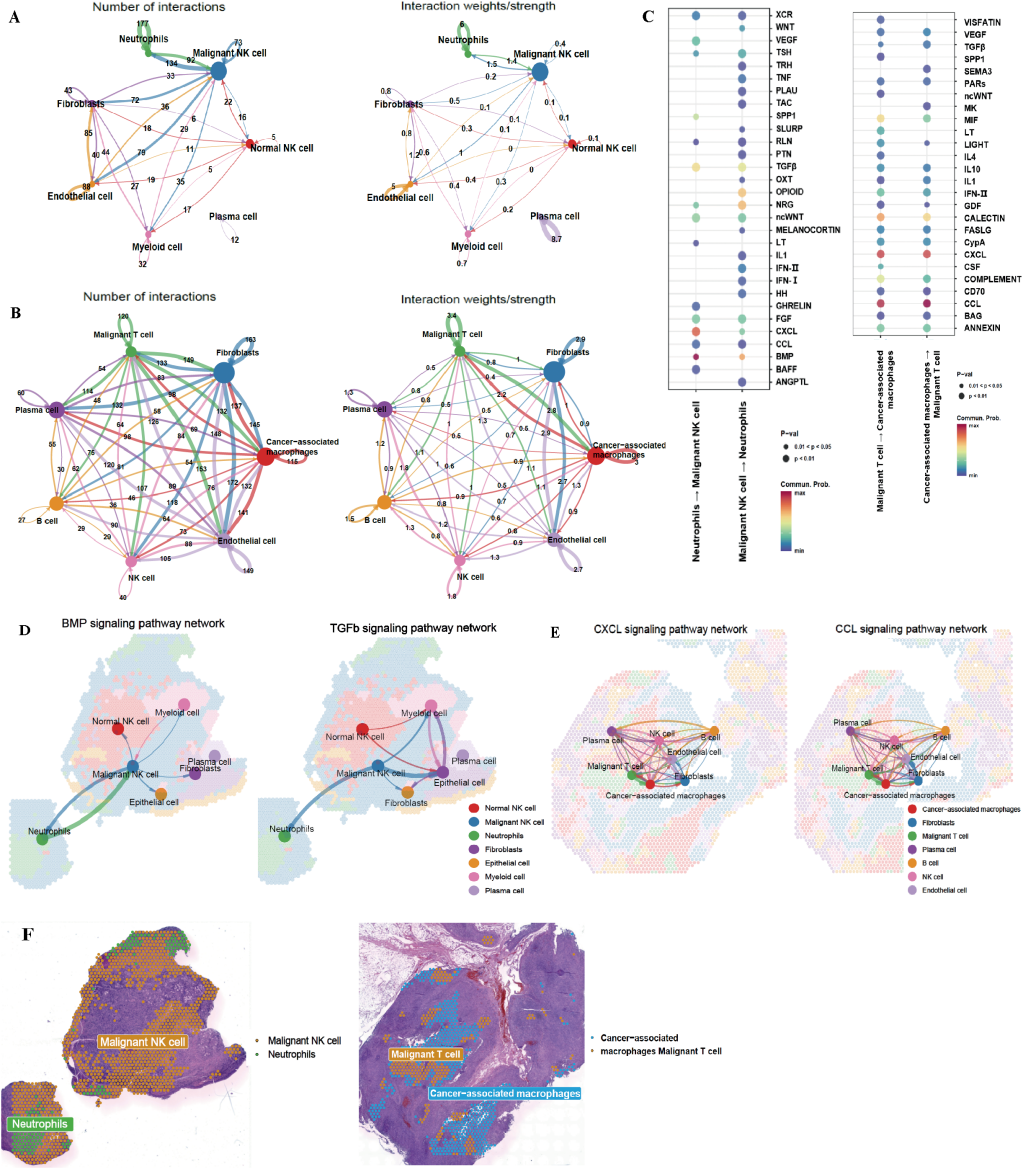
Cellular communication analysis. **(A, B)** Number and strength of interaction between among cellular clusters in the TME of ENKTL**(A)** and EBV^+^ nTNKL**(B)**. **(C)** KEGG enrichment of cell-cell communication signaling pathways. **(D)** Spatial interactions among different cellular clusters of ENKTL mediated by the BMP/TGF-β signaling pathway. **(E)** Spatial interactions among different cellular clusters of EBV^+^ nTNKL mediated by the CXCL/CCL signaling pathway. **(F)** Spatial distribution map of clusters. Left: Spatial localization of malignant cluster and neutrophils in ENKTL, Right: Spatial localization of malignant cluster and cancer associated macrophages.

To investigate the exact mechanisms of malignant cell–immune cell interactions in the tumor microenvironment, we mapped the incoming and outgoing interactions between all clusters to analyze cell–cell communication (**Figures 3A, B**; **Supplementary Figures S2A–D**). There were no significant differences between the roles of individual clusters in signal emission and reception (**Supplementary Figures S2E, F**). Interestingly, when both communication frequency and intensity were considered, the strongest interaction in ENKTL occurred between malignant cells and neutrophils, whereas in EBV^+^ nTNKL it was between malignant cells and tumor-associated macrophages. In addition, neutrophils in ENKTL showed strong interactions with plasma cells, while in EBV^+^ nTNKL malignant cells exhibited notable communication with epithelial cells.

We further analyzed pathways underlying malignant cell–neutrophil interactions in ENKTL and found predominant involvement of TGF-β and BMP signaling, both members of the TGF-β superfamily that signal via SMAD effectors (**Figure 3C**) (16). Spatial mapping revealed that BMP signaling was mainly active in malignant NK-cell (**Figure 3D**), consistent with previous reports showing that BMP promotes NK-cell proliferation, differentiation, and survival (17). In contrast, TGF-β signaling was enriched not only in epithelial cells but also in malignant NK-cell, with increased activity toward neutrophils, consistent with TGF-β–mediated neutrophil chemotaxis and polarization toward an N2 phenotype, thereby contributing to an immunosuppressive tumor microenvironment (18).

In EBV^+^ nTNKL, malignant cells and tumor-associated macrophages communicated primarily through CXCL and CCL chemokines, influencing multiple cell types within the tumor microenvironment (**Figures 3C, E**). CXCL and CCL chemokines activate downstream PI3K/AKT and MAPK signaling via their respective G protein–coupled receptors, thereby regulating migration, proliferation, and survival (19). Spatial analysis further showed that malignant NK-cell in ENKTL localized near neutrophils, whereas in EBV^+^ nTNKL they were surrounded by tumor-associated macrophages (**Figure 3F**). Within these signaling pathways, dominant ligand–receptor pairs were further characterized, and gene-level expression information for key ligand and receptor components is summarized in **Supplementary Tables S3**-**S6**.

### 3.4 Immune landscape reveals greater activation in EBV^+^ nTNKL than in ENKTL

**Figure 4:**
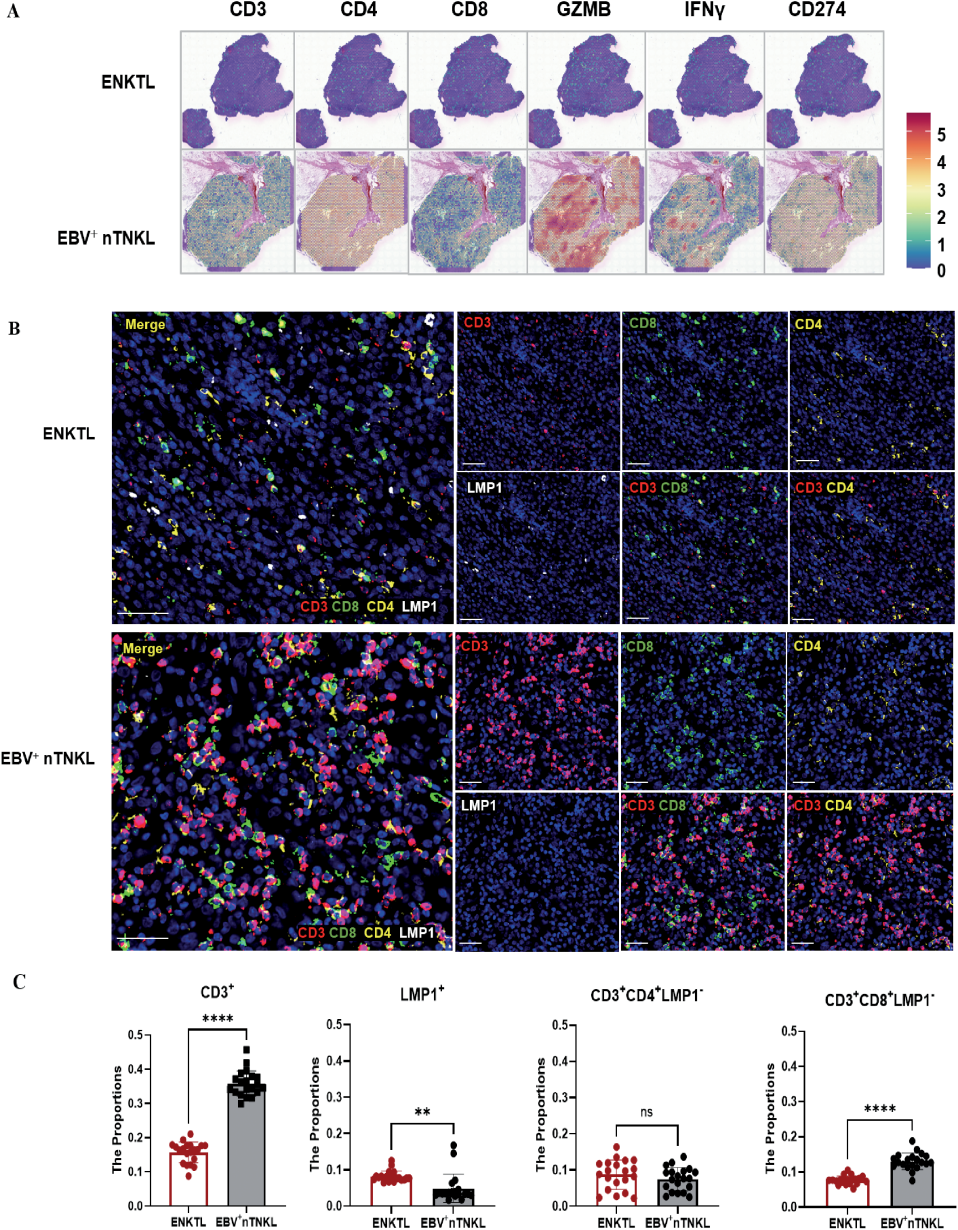
Immune landscape of ENKTL and EBV^+^ nTNKL. **(A)** Hematoxylin-eosin staining and spatial feature plots of marker genes in each sample. **(B)** MIF of tumor-infiltrating lymphocytes, with CD3, CD4, and CD8 labeling infiltrating T cells and LMP1 labeling tumor cells(Scale bar=50μm). **(C)** Quantitative analysis of TIL and LMP1^+^ malignant cell. MIF analyses were performed in an independent cohort of 10 ENKTL and 10 EBV^+^ nTNKL patients, with two randomly selected regions analyzed per sample.

To delineate the overall immune landscape of the two subtypes, we first quantified immune-related gene expression within spatial tumor regions. Compared with EBV^+^ nTNKL, ENKTL exhibited markedly lower expression levels, resembling an ‘immune desert’ phenotype (**Figure 4A**). This phenomenon may be explained by the relative genomic stability of EBV^+^ nTNKL compared with the higher genomic instability of ENKTL (20). In addition, widespread promoter hypermethylation and distinct EBV latent infection patterns in ENKTL may underlie the downregulation of immune-related genes and facilitate deeper viral integration.

**Figure 5:**
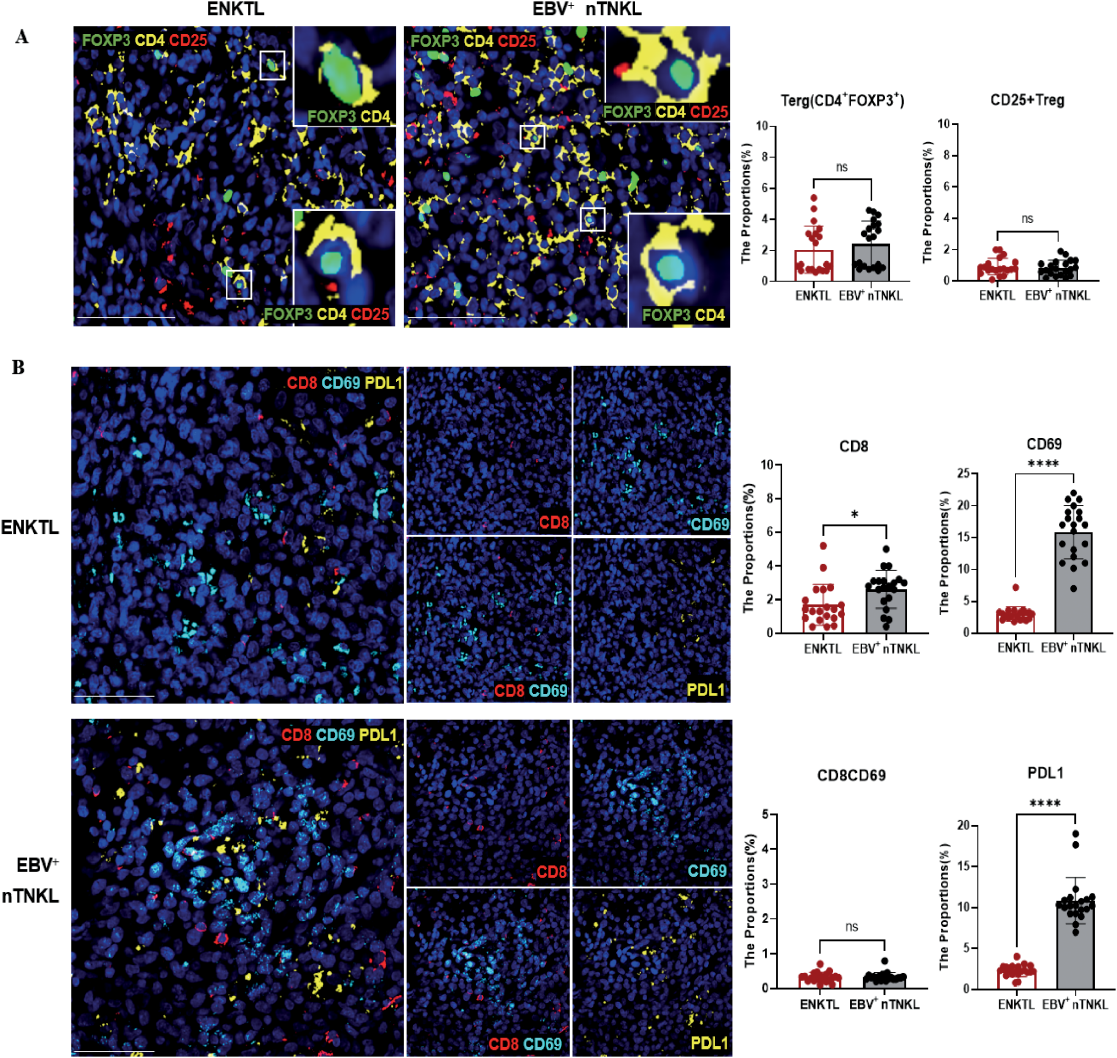
Immune ecosystem of ENKTL and EBV^+^ nTNKL. **(A)** MIF of Treg. **(B)** MIF of immune suppression and activation markers (CD8, CD69, PD-L1) (Scale bar=50μm). MIF analyses were performed in an independent cohort of 10 ENKTL and 10 EBV^+^ nTNKL patients, with two randomly selected regions analyzed per sample.

We next evaluated immune cell composition using mIF in an independent validation cohort. EBV^+^ nTNKL showed significantly higher densities of CD3^+^ T cells and CD3^+^CD8^+^ cytotoxic T cells than ENKTL, along with lower densities of LMP1^+^ tumor cells. No significant differences were observed in CD3^+^CD4^+^ T cells or Tregs (**Figures 4B, C**, **5A**). Moreover, EBV^+^ nTNKL demonstrated higher PD-L1 expression and increased CD69^+^CD8^+^ T-cell activity compared with ENKTL (**Figure 5B**). Together, these findings indicate that EBV^+^ nTNKL is characterized by a more active antitumor immune response compared with ENKTL.

### 3.5 Differential clinical and survival profiles between EBV^+^ nTNKL and ENKTL

**Figure 6:**
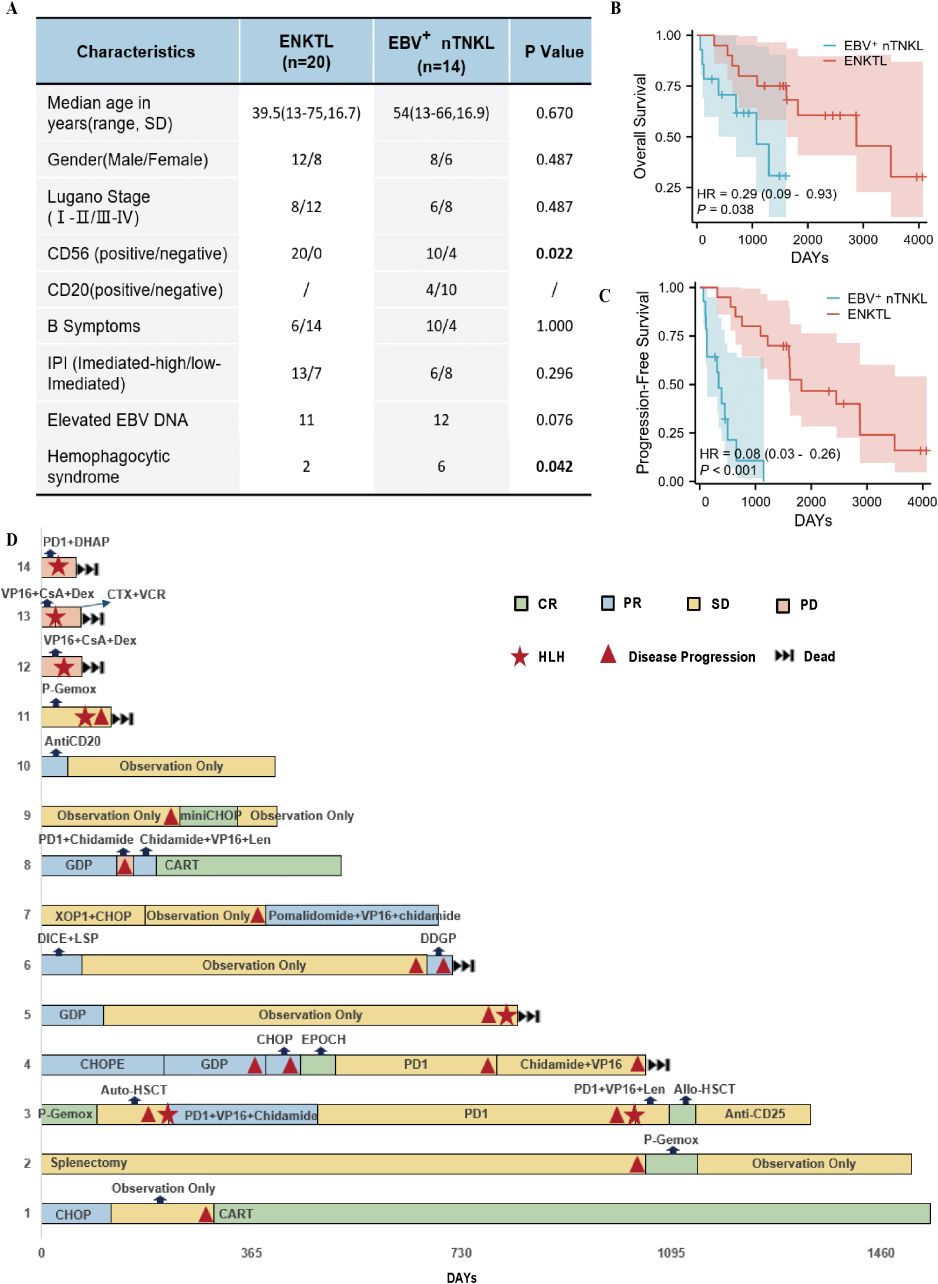
Clinical and therapeutic features. **(A)** Comparative analysis of clinical characteristics between ENKTL and EBV^+^ nTNKL. **(B, C)** Analysis of OS **(B)** and PFS **(C)** differences between ENKTL and EBV^+^ nTNKL. **(D)** Treatment overview of 14 patients with EBV^+^ nTNKL.

EBV^+^ nTNKL is extremely rare. We retrospectively reviewed all lymphoma cases diagnosed at the First Affiliated Hospital of Zhengzhou University from 2015 to the present and identified 14 patients who met the diagnostic criteria for EBV^+^ nTNKL, with ENKTL cases randomly sampled as controls for comparative analysis (**Figure 6A**). EBV^+^ nTNKL predominantly affected middle-aged to elderly males and showed CD56 positivity in 71.4% of cases (including focal or partial expression). Of note, CD20^-^positive background cells were detected in 28.6% of patients. In addition to lymphadenopathy, most patients presented with high-grade fever (71.4%), and 85.7% had elevated circulating EBV DNA levels. In, addition, the incidence of hemophagocytic lymphohistiocytosis was significantly higher in EBV^+^ nTNKL than in ENKTL (P < 0.05). The median OS and PFS for EBV^+^ nTNKL patients were 1,074 and 337 days, respectively, both of which were significantly inferior to those of ENKTL patients (**Figures 6B, C**).

In terms of treatment, given the limited cohort size and therapeutic heterogeneity, formal statistical analysis was not feasible; therefore, treatment courses and disease status for each patient were visualized in a swimmer plot to provide clinical insight into management strategies for this rare entity. To date, 7 of the 14 patients have died. Treatment regimens were heterogeneous and included T-cell lymphoma–oriented protocols such as GDP and P-Gemox, as well as B-cell lymphoma–oriented therapies including CD20 monoclonal antibodies and CHOP-like regimens, consistent with the presence of CD20-positive background cells in a subset of patients. Some patients also underwent splenectomy, autologous or allogeneic stem cell transplantation, and CAR-T-cell therapy. Among these, two patients treated with CAR-T-cell therapy achieved complete remission, with sustained responses lasting 1,245 and 320 days, respectively. In addition, two patients received PD-1 blockade monotherapy, achieving disease control for 552 and 280 days, respectively; one experienced a prolonged remission period, although both eventually relapsed (**Figure 6D**). These findings highlight the marked therapeutic heterogeneity of EBV^+^ nTNKL and suggest that approaches such as CD20-directed therapy, immunotherapy, and CAR-T may represent promising options in selected patients.

## 4 Discussion

Both ENKTL and EBV^+^ nTNKL are EBV-associated lymphoproliferative disorders (21). Persistent EBV infection profoundly remodels the tumor microenvironment, fostering malignant transformation and underscoring the need to elucidate these interactions for therapeutic advances (22). By integrating spatial transcriptomics with MIF, we provide an exploratory comparison of these subtypes in their native tissue context. Unlike bulk or single-cell approaches, spatial transcriptomics preserves tissue architecture and enables high-resolution mapping of malignant–immune cell interactions. Leveraging one of the largest cohorts worldwide, we assembled 14 EBV^+^ nTNKL cases, offering rare and valuable insights into this exceptionally uncommon entity, which is increasingly recognized as a distinct lymphoma subtype.

A notable observation was the apparent divergence in tumor burden and immune context, which may be shaped by distinct EBV latency programs. ENKTL, associated with latency II infection, exhibited higher malignant cell density and an ‘immune desert’ phenotype, consistent with restricted viral gene expression and immune evasion via EBNA2 downregulation (23, 24). By contrast, EBV^+^ nTNKL, linked to latency III, displayed an immune-active microenvironment enriched in CD8^+^ T cells and PD-1/PD-L1 expression, reflecting broad viral gene activity and stronger immunogenicity (25–27). These differences suggest biological distinctiveness of the subtypes and may provide a mechanistic basis for their differential responsiveness to immune checkpoint blockade (28, 29).

Lineage analysis further distinguished them: TCR sequencing confirmed the NK-cell origin of ENKTL and the T-cell origin of EBV^+^ nTNKL, consistent with their extranodal versus nodal presentations. Tissue context also shaped immune composition—ENKTL harbored neutrophil-enriched clusters, likely linked to chronic nasal inflammation, whereas EBV^+^ nTNKL contained abundant B cells consistent with its lymph node origin (30–32). These findings point to a possible role of anatomical niches in tumor–immune interactions. In previous work, we found that most ENKTL patients have a history of chronic rhinosinusitis (33–35). Intercellular communication analyses revealed additional subtype-specific features. In ENKTL, malignant cells engaged neutrophils via TGF-β and BMP signaling, promoting tumor proliferation and inducing an immunosuppressive N2 phenotype (36–38). In EBV^+^ nTNKL, malignant cells interacted predominantly with tumor-associated macrophages through CXCL/CCL–GPCR signaling, amplifying inflammatory responses. These results are in line with reports that GPCR modulation can restore antitumor T-cell activity in EBV^-^driven malignancies (39). At the genomic level, ENKTL exhibited marked instability, whereas EBV^+^ nTNKL showed relatively stable genomes but recurrent mutations in TET2, PIK3CD, and STAT3, alongside NF-κB and PD-L1 pathway activation (7, 40). Collectively, these observations suggest potentially distinct modes of immune regulation and molecular pathogenesis. Although based on single-patient spatial transcriptomic profiles, our findings suggest that subtype-specific biology may inform future combination strategies, which will require validation in larger cohorts before clinical translation.

Clinically, EBV^+^ nTNKL was observed to be more frequently complicated by hemophagocytic lymphohistiocytosis (HLH), likely reflecting its broad EBV antigen expression and hyperactivation of cytotoxic lymphocytes (41–44). In our cohort, uncontrolled HLH accounted for most deaths, underscoring the need for early recognition and intervention. Treatment responses were heterogeneous: conventional ENKTL- or PTCL-directed regimens yielded only transient benefit, whereas selected patients achieved durable responses with targeted or immune-based approaches. Anti-CD20 therapy was effective in CD20 positive cases, PD-1 blockade provided prolonged disease control in some patients, and CAR-T therapy induced complete and sustained remissions (45, 46). These proof-of-concept responses raise the possibility that immunotherapy and cellular therapy may hold promise, although validation in larger multicenter cohorts is required.

Several limitations of this study should be acknowledged. First, the spatial transcriptomic analysis was based on a single representative case from each subtype, which limits the generalizability of our findings; however, given the extreme rarity of EBV^+^ nTNKL, these data provide valuable exploratory insights. To partially address this limitation, key observations were validated in an independent cohort using MIF and supported by clinical data from 14 EBV^+^ nTNKL patients. Second, ENKTL and EBV^+^ nTNKL arise from distinct anatomical sites (nasal mucosa versus lymph node), and some observed differences in the tumor microenvironment may therefore reflect tissue-of-origin effects rather than intrinsic biological distinctions. Finally, treatment heterogeneity and limited sample size precluded robust statistical comparisons, and the findings should be interpreted with caution.

In conclusion, although ENKTL and EBV^+^ nTNKL share a common viral etiology, they appear to diverge in lineage origin, immune architecture, and clinical behavior. Our study provides preliminary insights into their immunologic and molecular features, highlights the potential influence of EBV latency and tissue context, and—by assembling one of the largest EBV^+^ nTNKL cohorts to date—offers rare but increasingly recognized insights into this understudied lymphoma. These findings may help lay the groundwork for future translational research and underscore the need for tailored therapeutic strategies, particularly incorporating immunotherapy and cellular therapy, for this challenging entity.

## Data Availability

The spatial transcriptomics sequencing data reported in this study have been deposited in the GSA-Human database under accession number HRA016726. All other data supporting the findings of this study are provided within the manuscript and its supplementary materials, or are available from the corresponding author upon reasonable request.

## Supplementary material

Supplementary Figure 1 Spatial transcriptomics results. **(A, B)** Reference genes dotplot of each clusters in ENKTL **(A)** and EBV^+^ nTNKL(B). **(C, D)** Heat maps of marker genes of different cell types in spatial transcriptomes and functional enrichment bars of different cell types in ENKTL **(C)** and EBV^+^ nTNKL **(D)**. **(E–G)** KEGG enrichment analysis of differentially expressed genes:(E)ENKTL vs CRS, **(F)** EBV^+^ nTNKL vs CRS, **(G)** ENKTL vs EBV^+^ nTNKL.

Supplementary Figure 2 Cellular communication analysis. **(A–D)** Number and strength of interaction between among cellular clusters in the TME of ENKTL**(A, B)** and EBV^+^ nTNKL**(C, D)**. **(E, F)** The outgoing and incoming strengths of each cell cluster in ENKTL **(E)** and EBV^+^ nTNKL **(F)**.
