# Supplementary figures and images for: Characterizing tumor microenvironment heterogeneity in EBV+ nTNKL vs ENKTL using spatial transcriptomics and MIF
Source: Front Immunol. 2026 Feb 11;17:1717844. doi: 10.3389/fimmu.2026.1717844 (PMC12933425; doi:10.3389/fimmu.2026.1717844)

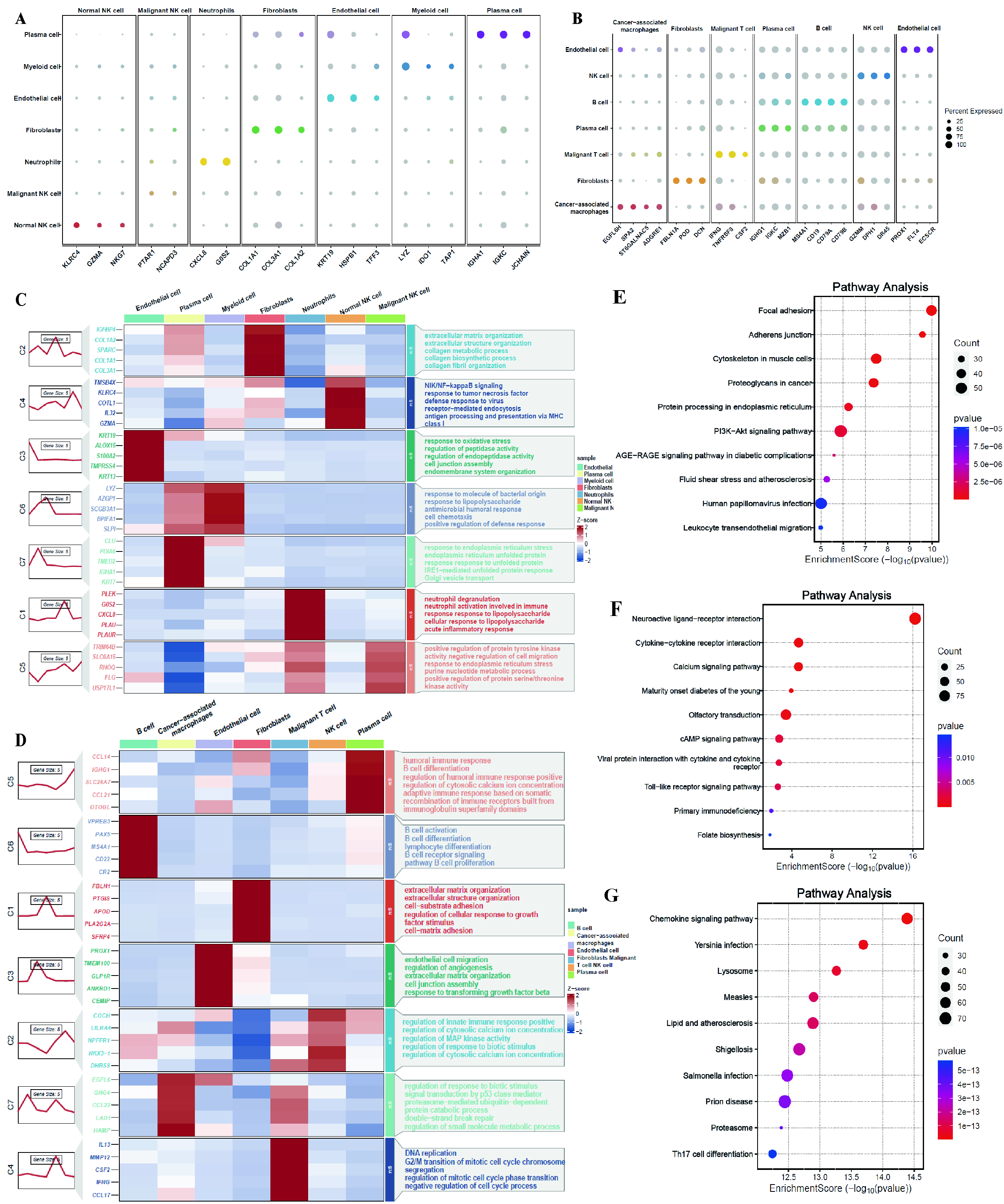

Supplement: Supplementary Figure 1 — Spatial transcriptomics results. (A, B) Reference genes dotplot of each clusters in ENKTL (A) and EBV+ nTNKL(B). (C, D) Heat maps of marker genes of different cell types in spatial transcriptomes and functional enrichment bars of different cell types in ENKTL (C) and EBV+ nTNKL (D). (E–G) KEGG enrichment analysis of differentially expressed genes:(E)ENKTL vs CRS, (F) EBV+ nTNKL vs CRS, (G) ENKTL vs EBV+ nTNKL. [file Image1.tif]

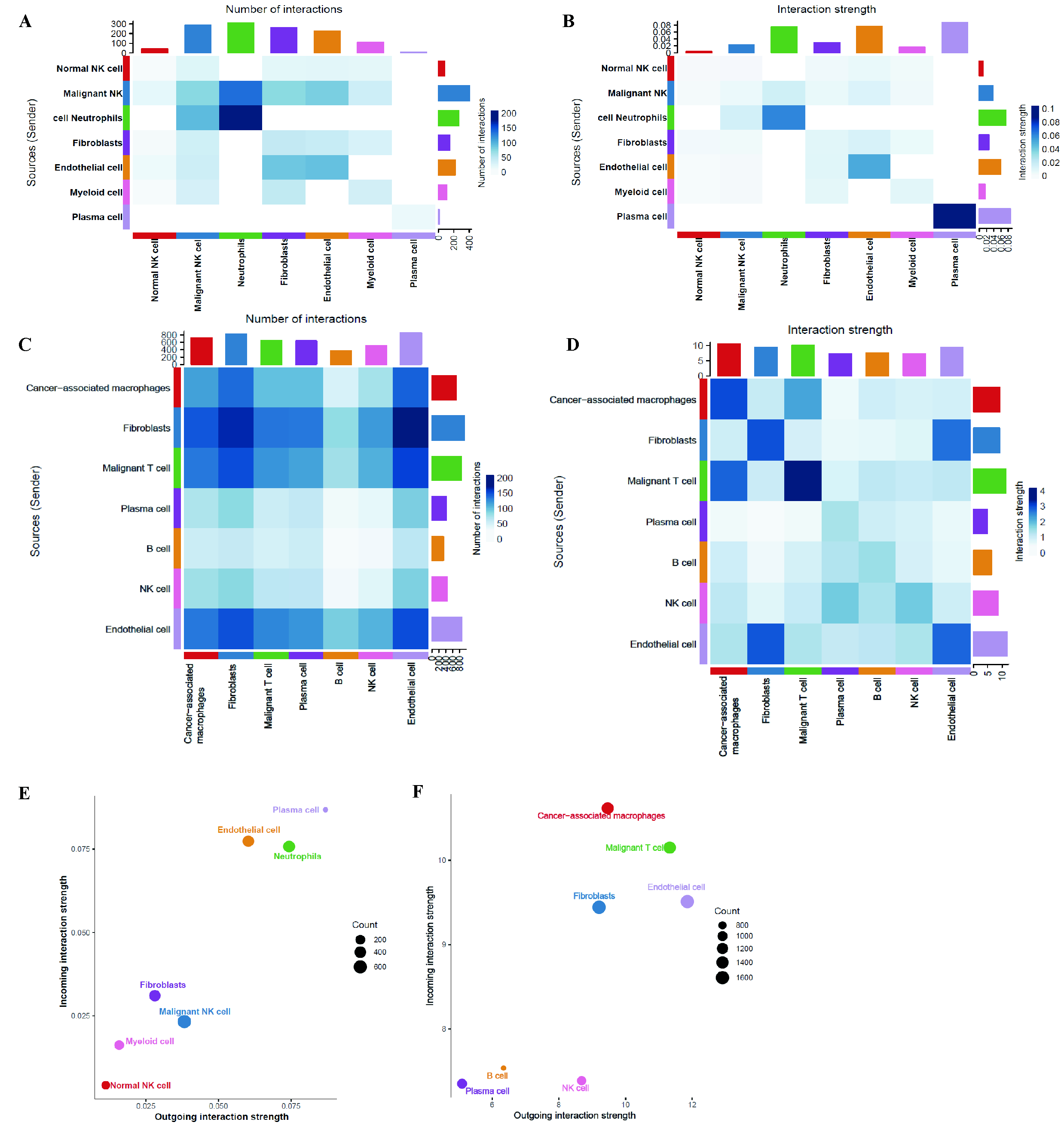

Supplement: Supplementary Figure 2 — Cellular communication analysis. (A–D) Number and strength of interaction between among cellular clusters in the TME of ENKTL(A, B) and EBV+ nTNKL(C, D). (E, F) The outgoing and incoming strengths of each cell cluster in ENKTL (E) and EBV+ nTNKL (F). [file Image2.tif]
